# Presumed Posterior Scleritis Complicated by Serous Retinal Detachment as a Possible Late Manifestation of COVID-19 Infection: A Pediatric Case Report

**DOI:** 10.7759/cureus.87284

**Published:** 2025-07-04

**Authors:** Jared J Tuttle, Shannon D Scarboro, Clio A Harper III, Taylor Lind, Ryan C Young

**Affiliations:** 1 Ophthalmology, University of Texas Health Science Center at San Antonio, San Antonio, USA; 2 Retina, Austin Retina Associates, Austin, USA

**Keywords:** case report, covid-19 infection, inflammatory eye disease, long covid, pediatric infectious disease, pediatric retina, pediatric scleritis, post-acute covid-19 syndrome, posterior scleritis, serous retinal detachment

## Abstract

A wide range of systemic and ocular symptoms have been associated with 2019 novel coronavirus (COVID-19) infection, including long-term alterations in inflammatory pathways. We describe a novel case of an 11-year-old female presenting with posterior scleritis and associated serous retinal detachment, occurring one year after confirmed COVID-19 infection. The patient presented with a one-day history of central scotoma following three days of progressive right eye pain, photophobia, conjunctival injection, and blurred vision. She reported two prior episodes of right eye pain, the first of which occurred in the setting of acute COVID-19 infection. Examination revealed clinical signs of posterior scleritis with serous retinal detachment. Laboratory work-up for infectious and autoimmune etiologies was unremarkable. The patient was treated with topical corticosteroids and nonsteroidal anti-inflammatory therapy, resulting in complete resolution of symptoms and restoration of visual acuity. Despite the atypical response, posterior scleritis remains the presumed diagnosis based on recurrent episodes and clinical findings. The temporal association with initial COVID-19 infection raises the possibility of an etiological role, though it remains unclear whether persistent viral antigens or a dysregulated immune response underlie the condition. This case introduces posterior scleritis as a potential delayed ocular manifestation of COVID-19 infection and highlights the need for further research into post-viral inflammatory responses affecting the eye. For clinicians, it emphasizes the importance of considering recent or remote COVID-19 infection in the differential diagnosis of posterior segment inflammation.

## Introduction

The 2019 novel coronavirus (COVID-19) has been associated with a diverse range of systemic and ophthalmic manifestations [1]. Following the acute infectious phase, prolonged sequelae of the disease have been identified and documented as “long COVID” or post-acute COVID-19 syndrome [2]. The lasting hypercoagulable and inflammatory changes caused by COVID-19 infection can result in diverse ocular manifestations in both the anterior and posterior segments [3]. To date, posterior scleritis has not been reported as a post-acute complication of COVID-19.

Posterior scleritis is characterized by inflammation of the sclera behind the ora serrata and represents a minority of scleritis cases [4]. Its disease sequelae can include serous retinal detachment, optic nerve edema, choroidal folds, and choroidal detachment [5]. The etiology includes a variety of infectious and autoinflammatory causes [6].

We report a case of an 11-year-old female developing presumed posterior scleritis complicated by serous retinal detachment one year following initial COVID-19 infection. This case provides further insight into the inflammatory effects of COVID-19 infection, furthering the discourse on late disease manifestations.

## Case presentation

An 11-year-old female presented with a one-day history of central scotoma following three days of worsening right eye pain, photophobia, conjunctival redness, minimal periorbital edema, and blurred vision. The pain was described as piercing, 7/10 in severity, and exacerbated with eye movement. The patient was otherwise healthy and denied any fever, cough, rash, joint pain or swelling, bug bites, tick exposure, or sick contacts. The patient denied the use of corticosteroids in any form, including oral, intranasal, or inhaled. The patient was prescribed topical tobramycin (0.3%) three times daily by her primary care provider, but the symptoms continued to worsen.

**Figure 1:**
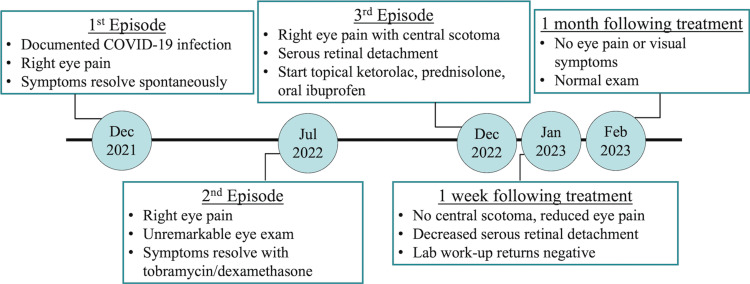
Timeline of Recurrent Episodes with Treatment Outcomes

Over the past year, the patient had experienced two prior episodes of eye pain (Figure 1). The first occurred one year prior in the setting of acute COVID-19 infection confirmed by reverse transcription polymerase chain reaction testing. The eye pain resolved spontaneously without medical intervention. The second episode of eye pain and vision loss occurred six months after the initial infection. She was seen by an ophthalmologist, who reportedly found an unremarkable eye exam. After a short course of tobramycin/dexamethasone eye drops, the patient’s symptoms resolved. 

Her medical history was notable only for attention-deficit/hyperactivity disorder, managed with dextroamphetamine-amphetamine. The patient denied use of tobacco products or alcohol. There was no family history of uveitis or any rheumatologic disorder. The patient did not report any known drug allergies.

**Figure 2:**
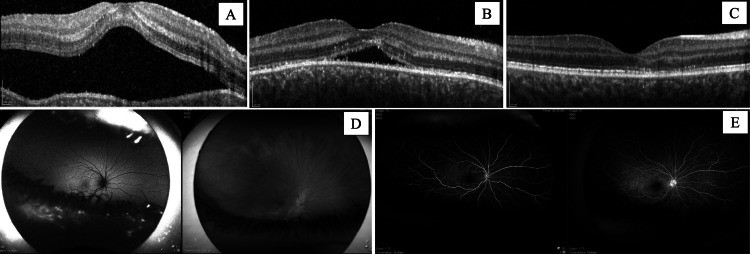
Imaging at Presentation and Follow-up Visits Optical coherence tomography of the right eye at presentation (A), one-week follow-up (B), and one-month follow-up (C) demonstrating serous retinal detachment with resolution. Fundus imaging of the right eye at presentation (D). Fluorescein angiography of the right eye demonstrating fluid accumulation at presentation (E). The image on the left was taken 20 seconds after fluorescein administration, and the image on the right was taken 4 minutes and 20 seconds after fluorescein administration.

On examination, the patient’s visual acuity was 20/60+2, which improved to 20/30-2 with pinhole testing in the right eye. The patient’s best-corrected visual acuity in the left eye was 20/20-1. The intraocular pressure was 12 mmHg in both eyes as measured by a handheld applanation tonometer (Tono-Pen). Dilated fundus exam revealed subretinal fluid and retinal pigment epithelium changes (Figure 2D). Optical coherence tomography and fluorescein angiography revealed a serous retinal detachment secondary to posterior scleritis (Figures 2A-2E). An extensive infectious and immunologic work-up was ordered (Table 1). Magnetic resonance imaging or B-scan ultrasound was not completed. The patient was prescribed topical prednisolone (1%) four times daily, topical ketorolac (0.5%) four times daily, and oral ibuprofen (400 mg) as needed. The patient was instructed to return to the clinic in one week.

**Table 1:** Summary of Infectious and Immunologic Work-up HLA: human-leukocyte antigen; Ig: immunoglobulin; IV: index value; IU: international units; N/A: not applicable; ANA: antinuclear antibody; ANCA: antineutrophil cytoplasmic antibody; TB: tuberculosis

Category	Test	Result	Reference Range (Units)	Interpretation
Hematologic Panel	Hemoglobin	13.4	11.0–15.5 (g/dL)	Within normal limits
	Hematocrit	39.5	33.0–45.0 (%)	Within normal limits
	White blood cell count	8.4	3.5–12.0 (k/μL)	Within normal limits
	Platelet count	369	200–500 (k/μL)	Within normal limits
	Beta-2 macroglobulin	1.0	0.6–2.29 (mg/L)	Within normal limits; does not suggest lymphoproliferative disease
Autoimmune/Genetic Markers	HLA-B27	Negative	Not detected	No genetic predisposition to spondyloarthropathy
	HLA-B51	Negative	Not detected	No genetic predisposition to Behçet’s disease
	Anti-nuclear antibody (ANA, IgG)	Negative	<1:40 (titer)	No serologic evidence of systemic autoimmunity
	Anti-neutrophil cytoplasmic antibody (ANCA, IgG)	Negative	<1:20 (titer)	No evidence of vasculitis
Infectious Disease Testing	Rapid plasma reagin (RPR)	Non-reactive	No antibodies detected	No evidence of active syphilis
	QuantiFERON-TB Gold Plus	Negative	<0.34 (IU/mL)	No latent or active TB infection
	Lyme disease antibody (IgM/IgG)	Negative	<0.9 (IV)	No evidence of Lyme disease
	Bartonella henselae antibody (IgG)	Negative	<1:128 (titer)	No evidence of Bartonella exposure or infection

At the one-week follow-up visit, the patient’s symptoms had greatly improved, and the central scotoma had resolved. The patient still endorsed occasional dull pain in the right eye. Examination revealed no conjunctival injection or periorbital edema. Best-corrected visual acuity was 20/20 in both eyes. The intraocular pressure was 21 mmHg in the right eye and 15 mmHg in the left eye as measured by Tono-Pen. Infectious and immunologic work-up results were negative (Table 1). Optical coherence tomography (OCT) imaging found a decreased serous retinal detachment (Figure 2B). The patient was instructed to continue topical prednisolone (1%) four times daily and reduce the frequency of topical ketorolac (0.5%) to two times daily. The patient was scheduled for a follow-up appointment one week later; however, the patient returned three weeks later.

At her follow-up visit one month after initial presentation, the patient reported complete resolution of symptoms. After completing the prescribed regimen one week prior to the visit, her symptoms had not returned. Visual acuity was 20/20 in both eyes, and intraocular pressure was 15 mmHg in the right eye and 17 mmHg in the left eye by Tono-Pen. OCT imaging revealed that the serous retinal detachment had completely resolved (Figure 2C).

## Discussion

Numerous ophthalmic findings have been associated with acute COVID-19 infection. Conjunctivitis is the most commonly reported manifestation, but retinal microvascular changes, optic neuritis, and uveitis have also been observed [7]. While most COVID-19-related uveitis cases involve the anterior segment, posterior segment involvement has been documented as well [8].

In the post-acute phase of COVID-19, a broad range of ocular complications have been described [3]. In a retrospective analysis of patients with a history of COVID-19 infection, Tohamy et al. observed a higher prevalence of hypercoagulability, neurological morbidity, uveitis, and central serous chorioretinopathy [9]. Compared to controls, patients with prior COVID-19 infection had higher levels of serum D-dimer and erythrocyte sedimentation rate, findings suggestive of ongoing inflammation. Other described conditions include ocular surface abnormalities, ocular neuropathic pain, central retinal vein occlusion, retinitis, and optic neuritis, among others [10-15].

COVID-19 infection has been associated with a number of autoinflammatory conditions [2]. Proposed mechanisms include direct viral invasion of ocular tissues and dysregulated immune responses [16]. Persistent viral antigens or molecular mimicry may drive the production of autoantibodies and sustained inflammation [3]. The incidence of autoimmune diseases such as Guillain-Barré syndrome and multisystem inflammatory syndrome in children has been linked to COVID-19 infection [17].

Scleritis may result from both infectious and autoinflammatory etiologies [18]. Therefore, an extensive work-up is recommended to rule out systemic causes [6]. Interestingly, posterior scleritis may occur more frequently in the pediatric population compared to adults [19]. Posterior scleritis has also been reported following COVID-19 vaccination [5,20], although our patient had no history of vaccination.

Given the patient’s recurrent eye pain, prominent serous retinal detachment, and fluorescein leakage on angiography, posterior scleritis remains the most likely diagnosis. However, in the absence of confirmatory imaging such as B-scan ultrasound (to identify the T-sign) or magnetic resonance imaging (to assess for scleral thickening), alternative diagnoses should also be considered (Table 2). Thus, posterior scleritis is the presumed diagnosis, with COVID-19 infection being a possible etiological factor due to temporal association with the first episode.

**Table 2:** Evaluation of Differential Diagnoses with Concordant and Discordant Findings OCT: optical coherence tomography; FA: fluorescein angiography; RPE: retinal pigment epithelium; VKH: Vogt-Koyanagi-Harada disease; CSCR: central serous chorioretinopathy; MIS-C: multisystem inflammatory syndrome in children; ESR: erythrocyte sedimentation rate; CRP: C-reactive protein; MRI: magnetic resonance imaging; ICGA: indocyanine green angiography; BP: blood pressure This table outlines the major differential diagnoses considered for an 11-year-old female presenting with unilateral eye pain, central scotoma, and serous retinal detachment. For each diagnosis, features consistent with the diagnosis (“supporting features”) and features that argue against it (“features that argue against”) are summarized based on clinical history, ophthalmic examination, and imaging findings.

Diagnosis	Supporting Features	Features That Argue Against
Posterior Scleritis	Unilateral eye pain exacerbated by movement; serous retinal detachment on OCT; leakage on fluorescein angiography; RPE changes on fundus exam; prior episodes	Absence of confirmatory imaging (e.g., B-scan or MRI to assess for scleral thickening or T-sign); no inflammatory markers (e.g., ESR, CRP) obtained; rapid clinical response to topical therapy without systemic immunosuppression
Early or Incomplete VKH Disease	Pediatric age; serous retinal detachment; associated viral trigger; negative serologies	Unilateral presentation (VKH is typically bilateral); no neurologic, auditory, or cutaneous findings (e.g., meningismus, tinnitus, vitiligo); no optic disc edema; rapid clinical response to topical therapy without systemic immunosuppression
Central Serous Chorioretinopathy (CSCR)	Serous retinal detachment without hemorrhage or vasculitis; history of stimulant use (which may elevate catecholamines)	Extremely rare in pediatric patients; absence of characteristic fluorescein angiography findings (e.g., smokestack or inkblot leakage); presence of ocular pain (not typical in CSCR); rapid resolution with anti-inflammatory treatment
Optic Disc Pit Maculopathy	Unilateral central vision loss; serous macular detachment may mimic posterior segment inflammation; can present in younger patients	No optic disc pit noted on exam or imaging; absence of characteristic schisis-like retinal changes on OCT; full resolution without persistent subretinal fluid; fundus imaging did not demonstrate congenital optic disc anomalies
Multisystem Inflammatory Syndrome in Children (MIS-C)	Temporal relationship to prior COVID-19 infection; ocular inflammation has been reported in MIS-C; recurrent nature may suggest systemic inflammatory dysregulation	No fever, mucocutaneous, gastrointestinal, or cardiovascular symptoms; absence of inflammatory marker testing (e.g., CRP, ferritin, D-dimer); no multiorgan involvement; isolated ocular presentation is atypical
Hypertensive Choroidopathy/Nephrotic Syndrome	Pediatric systemic disorders that can present with serous retinal detachment; steroid-responsive choroidal effusion may mimic scleritis	Blood pressure, renal function, and urine protein levels were not assessed; no clinical signs of hypertension or nephrotic syndrome (e.g., edema, fatigue); no known history of renal disease

While posterior scleritis often follows a more protracted course, this patient demonstrated rapid clinical improvement following the initiation of topical corticosteroids and nonsteroidal anti-inflammatory therapy. This favorable response may reflect early recognition and prompt treatment before structural damage occurred, a self-limited or idiopathic inflammatory process, or a transient immune reaction possibly triggered by prior viral infection. Additionally, pediatric patients may exhibit more robust tissue recovery and more efficient retinal pigment epithelium function than adults, contributing to quicker resolution of subretinal fluid.

The temporal relationship with prior COVID-19 infection raises the possibility that viral factors contributed to disease onset or recurrence. Although ocular manifestations of COVID-19 typically occur during the acute phase and are accompanied by systemic symptoms, delayed or episodic inflammation may result from residual viral antigens or dysregulated immune responses. A polymerase chain reaction test for SARS-CoV-2 was not obtained at presentation due to low clinical suspicion, and inflammatory markers (erythrocyte sedimentation rate, C-reactive protein) were not completed. Therefore, the proposed etiology may involve persistent viral antigens, immune-mediated inflammation, or a combination of both.

## Conclusions

This case highlights presumed posterior scleritis with serous retinal detachment as a potential delayed ocular manifestation of COVID-19 infection. It underscores the need for further investigation into post-viral immune responses and intraocular inflammatory pathways that may persist or be reactivated well after the resolution of acute infection. As understanding of post-acute sequelae of COVID-19 continues to evolve, heightened clinical and research attention to ophthalmic complications is warranted.

For clinicians, this case emphasizes the importance of considering recent or remote COVID-19 infection in the differential diagnosis of posterior segment inflammation, particularly in pediatric patients with recurrent symptoms. Although the presentation may appear sudden and vision-threatening, this case demonstrates that early recognition and timely anti-inflammatory treatment can result in rapid and complete visual recovery. Given the potential for delayed viral or immune-mediated ocular involvement, it may be prudent to include viral polymerase chain reaction (PCR) testing in the initial diagnostic work-up of posterior segment inflammation with unclear etiology, especially when there is a history of prior infection.
